# Relationship between edema and intracranial pressure following intracerebral hemorrhage in rat

**DOI:** 10.3389/fstro.2023.1155937

**Published:** 2023-03-29

**Authors:** Anna C. J. Kalisvaart, Natasha A. Bahr, Frederick Colbourne

**Affiliations:** ^1^Department of Psychology, University of Alberta, Edmonton, AB, Canada; ^2^Neuroscience and Mental Health Initiative, University of Alberta, Edmonton, AB, Canada

**Keywords:** edema, stroke, intracranial pressure (ICP), intracerebral hemorrhage (ICH), cerebral autoregulation

## Abstract

Elevated intracranial pressure (ICP) is a potentially fatal consequence of intracerebral hemorrhage (ICH). As the mass of the hematoma and regional edema builds, ICP rises and becomes increasingly variable acutely after stroke. High ICP may worsen cellular injury and edema by impairing local tissue perfusion, fueling a cycle that may ultimately cause fatality through ischemia and brain herniation. Time spent above an ICP of 20 mmHg often predicts a greater risk of death and disability following ICH. Compensatory mechanisms combat rising ICP. Classically, these include cerebrospinal fluid volume loss and cerebrovascular autoregulation, such as a reduction in the volume of venous blood. Additional mechanisms such as brain tissue compliance and skull volume compensation may also contribute. Compensatory compliance mechanisms are limited, and they vary by age and many other factors. Animal models of ICH are widely used to assess these variables and to gauge putative therapeutics. Most often those studies rely upon simple measures of edema, which may not accurately predict ICP data. Thus, we analyzed our past studies characterizing ICP, edema, and tissue compliance responses to striatal ICH in rat, including the collagenase (C-ICH) and whole blood models (WB-ICH). We found that both ICH models raised ICP, with greater effects in the C-ICH model, which may thus better reflect clinical findings of concern. Importantly, measures of edema, such as in the damaged hemisphere, on their own are not predictive of average or peak ICP response within either model, unless assessing across a very wide range of injury severities, or when including non-stroke animals. We caution against using edema data as a surrogate measure of mass effect and ICP following ICH.

## 1. Introduction

Intracerebral hemorrhage (ICH) is a frequently fatal stroke subtype characterized by the formation of a hematoma and localized swelling (edema) within the brain, taking up mass within the cranium (Wilkinson et al., 2021). The bony confines of the skull have limited capacity for rapid deformation, constraining intracranial volume (Benson et al., 2023); therefore, the added mass effect associated with ICH can result in elevated intracranial pressure (ICP) over days or even weeks following stroke onset, worsening brain injury and significantly increasing risk of death (Sykora et al., 2014). Mortality rates following ICH are generally high, with ~40–50% of patients dying within 30 days of their stroke (van Asch et al., 2010). Of these patients, an estimated ~50–75% die within the first 72 h following ICH (Sasaki et al., 2017), indicating the critical importance of better understanding the injury mechanisms that play out over this acute period. In the minutes, hours, and days following ICH onset, cerebral edema develops in the perihematomal region over cytotoxic, ionic, and vasogenic phases, each driven by distinct mechanisms that initially indirectly then directly influence ICP (Leinonen et al., 2018; Ironside et al., 2019).

Importantly, edema not only contributes to total intracranial mass effect, but also dictates the degree of tissue ionic dyshomeostasis, blood brain barrier (BBB) dysfunction, and perturbed cellular function, as documented in rodent models of ICH (Lee et al., 1997; Nadeau et al., 2019; Wan et al., 2023), with similar observations in patients (Aksoy et al., 2013). Progression of neuroinflammation and development of other secondary ICH complications such as fever (Malkinson et al., 1985b), seizures (Gabor et al., 1984; Vespa et al., 2007), and obstructive hydrocephalus also can severely disrupt ICP homeostasis (Lodhia et al., 2006; Wilkinson et al., 2021). Ultimately, such mechanisms are thought to drive ICP elevations and variability over time, with more severe deviations often associated with early neurological deterioration and coma (Sykora et al., 2014; Svedung Wettervik et al., 2020). Although several innate compensatory mechanisms provide some degree of ICP compliance [e.g., cerebral autoregulation, cerebrospinal fluid (CSF) flux, tissue compliance] in response to worsening ICH mass effect (Wilson, 2016; Bothwell et al., 2019; Kalisvaart et al., 2020; Ocamoto et al., 2021), these acute reserves are often compromised and quickly exhausted following severe ICH (Canac et al., 2020; Wilkinson et al., 2021). For instance, cerebral autoregulation normally ensures consistent cerebral blood flow (CBF) through homeostatic adjustments to cerebrovascular resistance *via* the coordinated action of myogenic, neurogenic, metabolic, and endothelial mechanisms (Cipolla, 2009; Armstead, 2016). Each of these respective cellular autoregulatory components can be disturbed in the setting of ICH, reducing the capacity for dynamic cerebrovascular control, and increasing likelihood of ICP decompensation (Armstead, 2016).

Elevated ICP independently contributes to secondary injury by worsening the ongoing disturbance to cerebrovascular integrity following ICH, and further impairing cerebral autoregulation (de-Lima-Oliveira et al., 2018). Distorted tissue architecture and microvascular compression may hinder adequate CBF, resulting in oxidative injury, energetic failure, and cell death (Lafrenaye et al., 2012; Guo et al., 2018). As a result, perihematomal edema and inflammation become progressively more severe, exacerbating a vicious cycle of worsening brain damage (Ironside et al., 2019). Accordingly, elevated ICP plays a key role in ICH mortality due to a combination of insufficient CBF, secondary cellular injury/death, and brainstem herniation (Ropper and King, 1984; Kalita et al., 2009; Al-Kawaz et al., 2021). Despite the ~50–70% of ICH patients who experience elevated ICP (Godoy et al., 2019), and the presumed importance of ICP in dictating early outcomes following moderate to severe ICH, it is very rarely measured directly in clinical or preclinical studies. This paucity of ICP data within ICH research can likely be attributed to several things, such as the fact that ICP assessment is typically invasive (both in patients and animal models), expensive, and produces widely varying estimates depending on the methodology, measurement location, and influence of anesthetics, among other parameters (Allen, 1986; Munakomi and Das, 2022).

Clinically, ICP is often measured using a CSF ventricular catheter and external fluid pressure sensor, or less often, through dedicated implantable ICP sensors (Evensen and Eide, 2020). Assessment of epidural ICP (e.g., placement of ICP sensor placed between dura and skull) is not used as often, owing to gradients in ICP and/or midline shift. Both methods require a neurosurgical approach. Non-invasive clinical ICP measures, on the other hand, are based on surrogate markers of ICP that are either not well-validated or are subject to major issues that limit their use (e.g., lack of continuous measurement, considerable inter-patient variability). Therefore, clinical ICP data is often not collected in the most severe of stroke patients, along with those with more modest bleeds where the presumed benefits of monitoring are outweighed by the risks incurred, such as deadly secondary complications like fever and infection (Dallagiacoma et al., 2022). Instead, the degree of cerebral edema, bleed size, and mass effect are radiologically assessed *via* computer tomography (CT) and/or magnetic resonance imaging (MRI), and these measures are collectively used as crude surrogate markers of mass effect severity (and therefore elevated ICP).

Regardless of how mass effect severity is evaluated, there are few treatments available to reduce pathologically elevated ICP or edema following ICH. Options range from benign strategies, such as changing patient head position or administration of non-invasive pharmacological treatment (e.g., hyperosmolar therapies, corticosteroids, and other agents that target the BBB), to more invasive neurosurgical options, such as decompressive craniectomy and/or hematoma evacuation (Ropper et al., 1982; Heuts et al., 2013; Takeuchi et al., 2013). Administration of hyperosmolar therapies, such as mannitol (Wang et al., 2015; Aminmansour et al., 2017; Han et al., 2022) or hypertonic saline (Riha et al., 2017; Shah et al., 2018; Han et al., 2022) have thus far shown limited efficacy in human patients, along with corticosteroids such as dexamethasone (Wintzer et al., 2020). Recent guidelines from the American Stroke Association (Greenberg et al., 2022) designate the use of hyperosmolar agents to treat elevated ICP/edema as class 2B (weak evidence for benefit after ICH), while use of corticosteroids are designated as class 3 (evidence of harm after ICH). Decompressive craniectomy is similarly designated as class 2B, likely in some part due to higher iatrogenic risk (Greenberg et al., 2022). Interestingly, although hyperosmotic therapies are typically transiently effective in reducing ICP levels by drawing water from the brain into the intravascular space, there is often a rebound effect where ICP can increase to levels higher than those observed pre-treatment, contributing to mixed patient outcomes (Nau, 2000; Grände and Romner, 2012). Additionally, though decompressive craniectomy lowers ICP, it does not necessarily improve intracranial compliance (Brasil et al., 2021). There is clearly yet much to uncover regarding the relationships between ICP, cerebral osmotic balance, edema, and their interplay with other intracranial compliance mechanisms. Thus, the promise of understanding how to best target non-invasive agents (such as hyperosmolar therapies) as a treatment for cerebral edema and elevated ICP remains an important clinical priority. Preclinical animal models of ICH are therefore vital in evaluating the timing, dosage, and administration of novel therapies for elevated ICP and edema moving forward.

Our recent systematic review of preclinical ICH literature reveals that while the majority (~60%) of studies assessed edema as an experimental endpoint, only < 1% of studies assessed ICP. This gap makes it difficult to gauge the clinical validity of the widely used rodent collagenase (C-ICH) and autologous whole blood (WB-ICH) models of ICH (Liddle et al., 2020), especially given the limited range of stroke severities that are typically used in order to navigate ethical and practical concerns (time and money) (Wilkinson et al., 2021). Cerebral edema is often evaluated in animal models *via* bulk assessment of the wet-dry brain tissue weight, or less commonly, *via* small animal imaging equipment (MacLellan et al., 2006, 2008), while ICP is assessed *via* implantable telemetry or *via* ventricular/parenchymal catheter and external pressure transducer (Harary et al., 2018; Wilkinson et al., 2021). Each of these methods have their own associated strengths and limitations. Assessment of wet-dry brain tissue weight as a measure of cerebral edema, for instance, is often confounded by the degree of serum extrusion from the hematoma, varying by the precise bleed location and amount of intact brain tissue in the sample (Williamson and Colbourne, 2017), but the low cost and ease of this method makes it a popular choice. Overall, the method and location of ICP assessment in preclinical studies (e.g., telemetric assessment vs. intraventricular or parenchymal catheter attached to external transducer, anesthetized vs. non-anesthetized animals) can impact measurements significantly (Wilkinson et al., 2021), making it difficult to compare across studies and to relate meaningfully to other endpoints, like edema.

Though very few rodent ICH studies assess both cerebral edema and ICP directly in the same animals, as mentioned, those who have done so have indicated that edema measurements do not relate well to ICP parameters at the same ICH severity, although it should be acknowledged that these were smaller studies (Hiploylee and Colbourne, 2014; Williamson and Colbourne, 2017; Kalisvaart et al., 2022). Additionally, if the degree of cerebral edema and magnitude of ICP elevations had a direct linear association, treatments that affect edema should also reduce ICP, and vice versa; however, preclinical evidence demonstrates that this is not always the case. For example, therapeutic hypothermia has been shown to reduce ICP with no effect on edema after ICH and other stroke subtypes (Murtha et al., 2015; John and Colbourne, 2016). Taken together, this evidence suggests that the degree of cerebral edema is not an adequate surrogate measure for ICP following ICH, at least as typically measured by preclinical ICH studies. Under normal circumstances, ICP and intracranial compliance dynamics are dictated by complex and bidirectional interactions between tissue volume homeostasis, osmotic regulation, dynamic cerebral autoregulation, and CSF flux (Wilkinson et al., 2021). These relationships are only further complicated by stroke. Components of the ICH injury cascade are also highly interrelated, thereby obfuscating individual contributions. For instance, vasogenic edema is worsened by post-stroke inflammation that further fuels BBB disruption, progressively increasing ICP and worsening edema (Zheng et al., 2016; Leinonen et al., 2018).

Therefore, as it stands, it is difficult to untangle the precise nature of the relationship between edema and ICP following stroke, yet an understanding of the links between these two factors remains a key aspect in developing improved predictive tools and therapeutics for patients. Here, we review the literature on regulation, timing, and dynamics of ICP and edema following rodent models of ICH, and we compare to the clinical scenario. We then retrospectively analyze data from five of our published studies that assess both ICP and edema following moderate to severe ICH, allowing for an exploration of these parameters and their relationships across ages and animal models of ICH with a greater degree of confidence thanks to a larger pooled sample size.

## 2. Background literature

### 2.1. Normal ICP and intracranial compliance regulation

Estimates of ICP in both humans and rats vary widely, depending on the method and location of assessment, and are often confounded by anesthetics. In humans, ICP is thought to range from 5 to 15 mmHg under normal circumstances (Czosnyka, 2004), while in adult rats, ICP ranges from 4 to 5 mmHg, at least in our hands (Silasi et al., 2009; Williamson and Colbourne, 2017; Williamson et al., 2018, 2019). Across the experimental literature, these values range as wide as 4–12 mmHg in conscious animals (Jiang and Tyssebotn, 1997; Chowdhury et al., 2013; Guild et al., 2015; Eftekhari et al., 2020), or 7–20 mmHg in anesthetized animals (Malkinson et al., 1985a; Barth et al., 1992; Zwienenberg et al., 1999; Goren et al., 2001; Bothwell et al., 2021), though these estimates may vary depending on the anesthetic used. For instance, volatile anesthetics such as isoflurane tend to increase ICP, thanks to their vasodilatory effect (Li et al., 2014), while barbiturates or sedative-hypnotic anesthetics may decrease ICP by reducing both systemic blood pressure and cerebral metabolic demand (Roberts and Sydenham, 2012; Oddo et al., 2016). Anesthetic choice is therefore a critical aspect to consider when using rodent models to evaluate ICP dynamics, as this can vary considerably across studies. Our recent review of preclinical ICH research demonstrated that roughly a third of preclinical ICH studies use chloral hydrate (a sedative-hypnotic), for example, while a quarter use pentobarbital (a barbiturate), and a sixth of studies use isoflurane (a volatile anesthetic) (Liddle et al., 2020).

**Figure 1:**
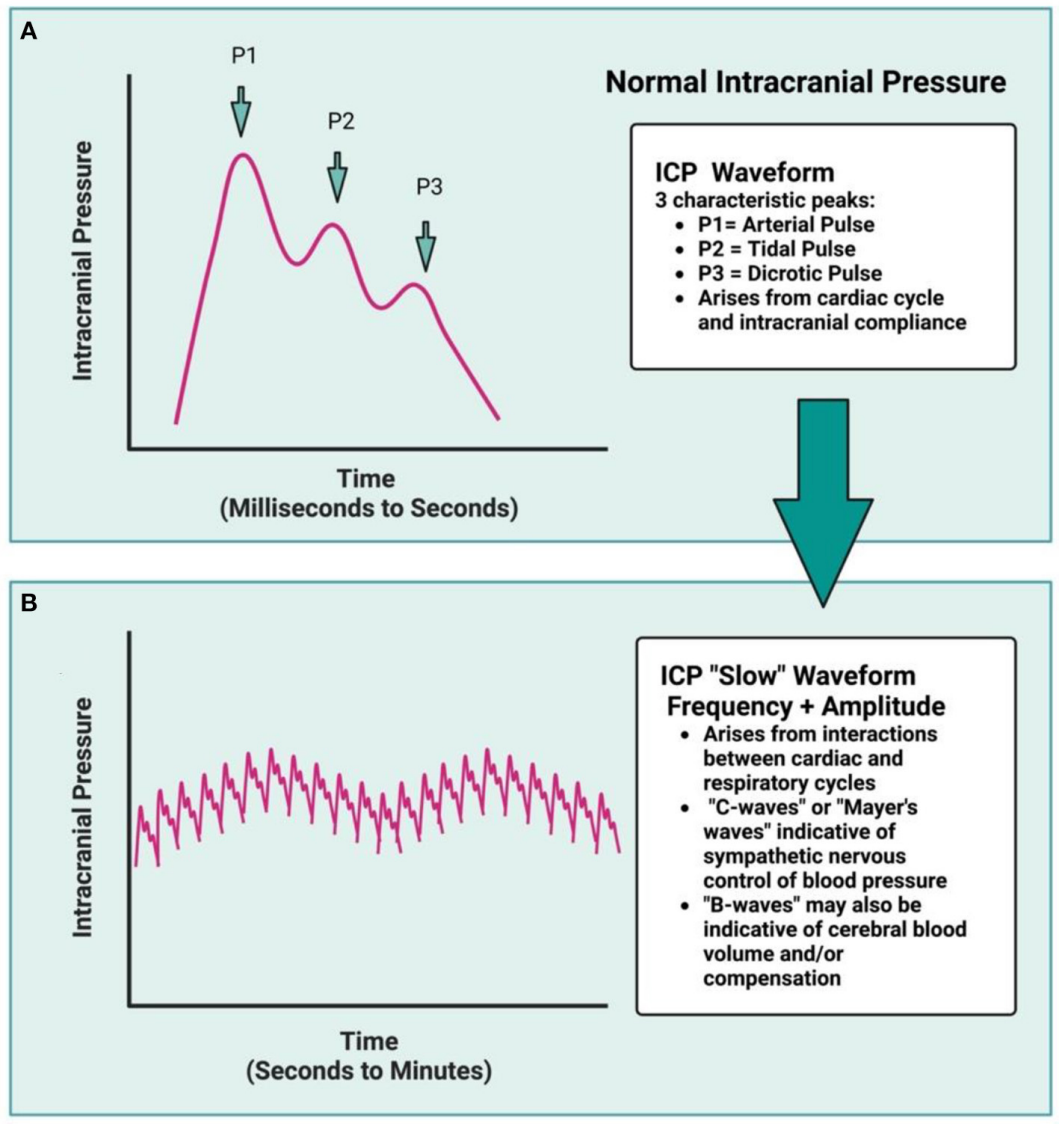
The ICP waveform is synchronous with arterial pulse and contains three distinct peaks **(A)**: P1, relating to the systolic percussion wave generated by left cardiac ventricular ejection, followed by P2, generated by a slightly later systolic tidal pressure wave, which arises from arterial elastance. Lastly, P3 correlates with the closure of the cardiac aortic valve and the dicrotic pulse (driven by cardiac systole and diastole). Under normal conditions, the amplitude of P1 > P2 > P3, but under decompensated state (such as following ICH), the amplitude of P2 may be greater than P1, reflecting reduced intracranial compliance. Over time, the cardiac cycle generates the ICP waveform, while the interaction between the cardiac and respiratory cycles dictates the amplitude and frequency of the ICP waveform over time **(B)**, resulting in characteristic “ICP slow waves.” The respiratory cycle influences ICP *via* changing intrathoracic pressure as a result of breathing, and is synchronized with central venous pressure, at least under normal conditions. In a decompensated state, this respiratory rhythm is no longer distinct as ICP rises. Rodent data typically are not of high enough resolution for waveform analyses to be done, nor are additional variables (blood pressure, heart rate, respiration) typically measured concomitantly.

Normally, ICP is the sum product of venous, arterial, and cerebrospinal fluid (CSF) pressures, as dictated by ongoing maintenance of cellular activity (Czosnyka, 2004; Bothwell et al., 2019). This necessitates tight regulation of each component to maintain adequate CBF, while also limiting ICP to a homeostatic range. The characteristic ICP waveform therefore remains largely similar over time, with rhythmic fluctuations that reflect the balance of changing arterial, venous, and CSF pressures (Figures 1A, B) (Fan et al., 2008; Frigieri et al., 2021). Ideally, changing arterial pulse pressures are buffered by venous outflow and CSF production, circulation, and drainage- this keeps ICP levels within a well-regulated range (Figures 1A, B). Changes in systemic mean arterial blood pressure (MAP) arise from cardiac output and the respiratory cycle, along with the ongoing interaction between blood pressure levels, partial pressures of CO_2_ and O_2_, serum osmolarity, and peripheral resistance, among others (Wilson, 2016; Svedung Wettervik et al., 2020). Cerebral perfusion pressure (CPP), or the pressure required to maintain CBF, is calculated by subtracting ICP from MAP (Wilson, 2016). The cerebral vasculature is protected from fluctuating CPP *via* static and dynamic cerebral autoregulation, ensuring adequate microvascular perfusion (Claassen et al., 2021).

Relatedly, osmotic balance, cell volume, and volume of extracellular space are also normally tightly regulated in the brain, as these parameters are closely tied to neuron membrane potential, capacitance, and propagation of electrical signals. Cell volume is also inherently linked to metabolic efficiency, function, and health (Danziger and Zeidel, 2015). Therefore, to protect cellular functionality, the cerebral osmotic balance is primarily regulated by expression of aquaporin-4 channels (AQP4) within astrocytes at the BBB and meninges under normal circumstances (Danziger and Zeidel, 2015). Expression of AQP4 localized to pial and vascular astrocytic projections affords precise bidirectional control of water influx and efflux, ionic homeostasis (e.g., potassium levels) and glymphatic waste removal (Chen et al., 2021). Additionally, both primary and secondary transport channels like the Na^+^/K^+^ exchanger or the Na^+^/K^+^/Cl^−^ co-transporter (NKCC1) play a key role in maintaining the ionic and osmotic balance within the brain (Stokum et al., 2016). Lastly, expression of transcellular and paracellular proteins, such as caveolins or those that make up tight junctions, also play an important role in cellular and BBB integrity/permeability under both normal and pathological circumstances (Hladky and Barrand, 2016; Kaya and Ahishali, 2020). The mechanisms outlined in this paragraph are well-reviewed elsewhere in more detail by Hladky and Barrand (2016), Kaya and Ahishali (2020), and MacAulay (2021). These factors are also important in maintaining ICP homeostasis. Each of the above factors involved in ICP homeostasis under normal circumstances are in turn influenced by numerous patient parameters, such as age, sex, level of physical activity, and health status (Czosnyka et al., 2005; Santos et al., 2017; Zanello et al., 2018; Gogniat et al., 2022).

### 2.2. Pathological ICP, edema, and intracranial compliance

Following an ICH, each component involved in ICP regulation and maintenance are compromised in some fashion, with potentially fatal consequences. Indeed, in human patients, an increase in ICP variability along with a greater amount of time spent above 20 mmHg are both significantly associated with a greater risk of death or disability following ICH, emphasizing the importance of better understanding intracranial compliance as a treatment target (Tian et al., 2013; Al-Kawaz et al., 2021). In the early hours during and following acute bleeding, cerebrovascular autoregulation, CSF and glymphatic circulation, and cellular volume homeostasis become dysregulated due to the hematoma, cerebral edema, and associated mechanical damage (Hoff and Xi, 2003; Chen et al., 2015; Faragó et al., 2016; Bothwell et al., 2019; Jiang et al., 2022). Following ICH, edema develops *via* three distinct mechanistic phases: (1) cytotoxic edema, (2) ionic edema, and (3) vasogenic edema (Hoff and Xi, 2003; Stokum et al., 2016; Wan et al., 2023). Cytotoxic edema is thought to be driven primarily by a combination of toxic blood break-down products and energetic failure (Liang et al., 2007), but note that this cellular swelling characteristic of cytotoxic edema is distinct from parenchymal tissue swelling, and simply represents a rearrangement in cerebral water content (Ironside et al., 2019). This can result in cell death if severe enough (Liang et al., 2007). The intracellular ionic shift characteristic of cytotoxic edema depletes extracellular stores, setting the stage for ionic edema, the second stage of edema formation.

Ionic edema occurs in the hours following stroke onset (Ironside et al., 2019; Wan et al., 2023). Given that the BBB largely remains intact in the hours following ICH, the ionic displacement triggered by cytotoxic edema creates a driving force for both ions and water from the intravascular and possibly cerebrospinal fluid (CSF) compartments (Chen et al., 2021), effectively increasing water mass and hydrostatic pressure within brain tissue. Edematous tissue swelling is generated as a result, further worsening ionic dyshomeostasis in the perihematomal region (Yang et al., 1994; Aksoy et al., 2013; Nadeau et al., 2019). The third phase is formation of vasogenic edema, which occurs hours and days following ICH onset as the neuroinflammatory response to cerebral injury sets in, and BBB integrity is lost (Zheng et al., 2016). Extravasation of plasma proteins into the brain is facilitated by “leaky” vasculature, further driving intracellular water movement and facilitating the spread of cerebral edema (Zheng et al., 2016). Severe ionic and vasogenic edema are more likely to cause elevated ICP (Leinonen et al., 2018), often resulting in local tissue distortion (e.g., midline shift), and placing some regions at risk of additional injury, depending on structural characteristics of surrounding cerebral architecture (Brock et al., 1975; Lafrenaye et al., 2012; McKeown et al., 2022). The spread and development of edematous tissue in regions surrounding the hematoma continues over the acute period post-ICH in rodents (12–72 h) (Lee et al., 1997; Hiploylee and Colbourne, 2014; Williamson and Colbourne, 2017), but this can persist longer in patients with severe ICH (Gebel et al., 2002; Venkatasubramanian et al., 2011). It is also important to note that the phases of edema formation within brain tissue are not necessarily temporally distinct, as they may occur in parallel to each other over tissue space.

Over this same period, the summation of these local pressure perturbations begins to manifest as an increase in global ICP levels, and in response, numerous compensatory mechanisms begin to engage (Papo et al., 1979; Chambers et al., 2001; Wilkinson et al., 2021). It is not entirely clear which mechanisms are prioritized first in response to rising pressure levels, but a combination of changes to local cerebral autoregulation, altered CSF circulation and drainage (e.g., upregulated AQP4 expression), and tissue volume adaptations (distal tissue shrinkage) all occur in attempt to maintain ICP homeostasis. The extent to which each mechanism is activated is likely dependent on the responsivity of perturbed tissue (Kalisvaart et al., 2020), the degree to which cerebral autoregulation remains intact (Ma et al., 2016), and how routes of CSF flux are affected (e.g., larger bleeds may block outflow, worsening ICP elevations, while smaller bleeds may leave drainage/clearance routes more intact) (Mokri, 2001; Koh et al., 2007; Bothwell et al., 2019; Vinje et al., 2020). The compliance response is also dependent on the patient context; age and hypertension are associated with worse control of CSF and vascular autoregulatory dynamics for example (Czosnyka et al., 2005; Kohn et al., 2015; Lindesay et al., 2016; Xu et al., 2017; Zhao et al., 2018), while age-related atrophy appears to alter intracranial compliance dynamics following ICH (Kalisvaart et al., 2022).

There is evidence for the existence of a centralized sympathetic baroreflex activated rapidly by increasing ICP (Schmidt et al., 2018), similar in principle to pressure-sensitive areas identified in the brainstem (McBryde et al., 2017). This mechanism normally operates to protect cerebral perfusion from modest increases in ICP associated with changing positions (upright to supine, for example) in normotensive individuals (Ropper et al., 1982). Larger ICP elevations (e.g., such as following stroke or brain injury) may then result in sympathetic over-activation (Schmidt et al., 2018), especially in a hypertensive state (Valensi, 2021). The associated vasoconstriction causes an increase in MAP to combat cerebral hypoperfusion and to maintain CPP. In some cases, this sympathetic innervation is not adequately balanced by parasympathetic innervation of cerebral vasculature (Dorrance and Fink, 2015; Valensi, 2021), often due to hypertension-associated loss of autoregulatory capacity, or injury-induced vasospasm. The combined result of these factors is thought to result in localized hypoperfusion, worsening cell death and edema while further increasing ICP and microvascular compression, and triggering a run-away sequence of intracranial decompensation in severe ICH patients (Ropper and King, 1984; Sykora et al., 2014). Patients at imminent risk of fatality may display Cushing's triad, or increased MAP, bradycardia, and irregular respiration (Marshman, 1997; Kalmar et al., 2005). The extent to which this occurs following moderate to severe ICH is likely dependent on mass effect volume (e.g., hematoma and edema), along with remaining functionality of ICP compliance reserves (e.g., tissue compliance, increased venous and CSF outflow) on a case-by-case basis.

Despite the logical assumption that global edema levels would be representative of some portion of overall mass effect, bulk assessment of edema [e.g., wet-dry weight method, varying CT and MRI scan parameters (Zhang et al., 2022)] does not always relate well to ICP measurements following strokes of a similar severity. There are likely several reasons for this disparity, such as variable methodological and operational definitions, measurement sensitivity, and study cohorts of a limited size. Though on a local level, it is entirely likely that the three mechanistic phases of edema development establish hydrostatic pressure gradients which then globally contribute to ICP levels, it is difficult to capture this relationship given the spatial and temporal sensitivity of current methods. Many edema measurements are taken as single assessments rather than *via* dynamic assessment over time, making it difficult to determine their relationship to the numerous possible temporally dependent ICP parameters. Here, we pool data from five of our past studies to assess how well edema reflects ICP, but also how well ICP responses following ICH rodent models reflect what is seen clinically, speaking to their validity.

## 3. Methods

### 3.1. Retrospective analysis design

Data was extracted from experiments within five of our previously published studies measuring ICP following ICH in male rats, using either the C-ICH or WB-ICH model (Hiploylee and Colbourne, 2014; John and Colbourne, 2016; Williamson and Colbourne, 2017; Williamson et al., 2019; Kalisvaart et al., 2022). These measurements were collected in awake freely moving animals through use of company-calibrated telemetry blood pressure transmitters connected to an epidural cannula (Data Sciences Int., probe model PA-C10), as previously documented (Williamson et al., 2018). Values were also corrected by offset readings taken before and after removal, as recommended. All experimental procedures were originally approved by the University of Alberta Biological Sciences Animal Care and Use Committee (protocol #960) prior to data collection, adhering to the Canadian Council of Animal Care guidelines. In the included experiments, ICP data was collected following sham procedure (surgery without an ICH), severe C-ICH, or WB-ICH (at doses of 100, 130, or 160 μL of autologous blood within groups, denoted as WB-100, WB-130, and WB-160, respectively). Consistent surgical methods (e.g., dose of isoflurane anesthesia, procedure for stereotaxic injection of collagenase or autologous blood) were used across studies, each modeling a moderate to severe stroke severity. Animals were randomized to sham or ICH procedure in all cases. As the final endpoint, each of these experiments also assessed edema by calculating regional brain water content percentages (BWC%) *via* the wet-dry weight method.

Four out of the five studies used young adult male animals (~300–500 g, 3–5 months of age; groups are denoted by C-ICH-Adult, WB-ICH-Adult, Sham-Adult), recording ICP over 72 h post-procedure, followed by euthanization and evaluation of BWC% as a measure of cerebral edema (Hiploylee and Colbourne, 2014; John et al., 2015; Williamson and Colbourne, 2017; Williamson et al., 2019). The other study used middle aged males (~650 g, 9–11 months of age; groups denoted by C-ICH-Aged, Sham-Aged), recording over 24 h, followed by euthanization and evaluation of BWC% (Kalisvaart et al., 2022). Different survival times were used in these studies for experimental reasons (e.g., aged rats in the Kalisvaart et al., 2022 study were euthanized at 24 h post-ICH as this is the timing of maximal tissue compliance, for instance). Pooling across studies (after original study exclusions, Table 1), data from 21 C-ICH-Adult animals, 24 WB-ICH-Adult animals, 15 Sham-Adult animals, 10 C-ICH-Aged animals, and 10 Sham-Aged animals were included in the present analyses (prior to analysis exclusions, see Results). Data from C-ICH and WB-ICH animals were compared to pooled shams for ICP and BWC% analyses, separating by age when appropriate; comparisons were not drawn directly between models.

**Table 1:** An outline of studies, experiments, and experimental groups included in the present retrospective analyses.

**Experiment**	**Experimental number**	**Groups**	**Age/weight**	**Sex**	**Survival**	**Edema**
John and Colbourne (2016)	Experiment 1	5 SHAM-Adult 5 C-ICH Adult	~13–24 weeks ~300–600 g	Male	72 h	Ipsilateral striatum
									Contralateral striatum
									Ipsilateral cortex
									Contralateral cortex
									Cerebellum
Hiploylee and Colbourne (2014)	Experiment 3	6 SHAM-Adult 8 WB-100-Adult 8 C-ICH-Adult	~ 3–5 months ~350–500 g	Male	72 h	Ipsilateral striatum
									Contralateral striatum
									Ipsilateral cortex
									Contralateral cortex
	Kalisvaart et al. (2022)			Experiment 2	10 SHAM-Aged 10 C-ICH-Aged	~12 months ~650 g	Male	24 h	Ipsilateral hippocampus
								Contralateral hippocampus
								Ipsilateral striatum
								Contralateral striatum
Williamson et al. (2018)	Experiment 5	8 C-ICH-Adult	~8–12 weeks ~250–350 g	Male			72 h	Ipsilateral hemisphere
							Contralateral hemisphere
							Cerebellum
Williamson and Colbourne (2017)	Experiment 3	5 SHAM-Adult 5 WB-100-Adult 5 WB-130-Adult 6 WB-160-Adult		~ 8–12 weeks ~250–350 g	Male	72 h	Ipsilateral hemisphere
										Contralateral hemisphere
									Cerebellum

Demographic data of each experimental group, along with specifics on edema endpoints collected in each case are also presented.

### 3.2. Intracranial pressure analyses

Raw data from each of the five included studies was first examined for artifact (e.g., values that exceed the previous hour's average by ≥5x, and did not persist over time) by an experimenter blinded to group allocations. On average, this was only 0.26% of the raw data points within each animal included. Data from WB-ICH and C-ICH groups were assessed separately, with average and peak 24 h and 72 h ICP calculated and compared to age appropriate controls (average and peak 1-min and hourly ICP values over each epoch are outined in Table 2). In order to assess ICP spiking events, data from each study was analyzed using a Python script (Kalisvaart et al., 2022) to identify (a) when ICP increases ≥ 10 mmHg for at least 3 min compared to the preceding 60-min average (e.g., “disproportionate increase in ICP”, or “DIICP event”) and (b) when ICP increased to values ≥ 20 mmHg for at least 3 min, and the preceding 60-min average was also ≥20 mmHg (e.g., “raised ICP” or “RICP” event). These criteria were determined by clinical definitions of intracranial hypertension. Once each DIICP or RICP event was identified, ICP data over the event duration was extracted, along with the preceding 60 min of data (pre-event baseline). Pre-event baseline data was first averaged within animals who experienced events. Corresponding “sham-equivalent” values were then determined by averaging sham data over the recording period, extracting 60 min of data corresponding to the timing of each ICH pre-event baseline. The pre-event baseline data was then split into four 15-min epochs, and the slope of ICP change over time was calculated for each epoch, comparing ICH and “sham-equivalent” values over each epoch in order to assess local ICP variability. Average and peak ICP over each DIICP and RICP event was also determined, along with event duration, timing, and number of events per animal within 24 and 72 h timeframes. Data processing was done using Microsoft Excel (v.16.61) and Anaconda Python v.3.9 (Spyder Package v.5.15).

**Table 2:** A characterization of average and peak 1-min and hourly ICP over 24 and 72 h epochs, split both across original studies, as well as compiled into the groups that were compared in the present data analyses.

**Experiment**	**Groups**	**1-min average ICP (24 h)**	**1-min average ICP (72 h)**	**1-min peak ICP (24 h)**	**1-min peak ICP (72 h)**	**Average hourly ICP (24 h)**	**Average hourly ICP (72 h)**	**Peak hourly ICP (24 h)**	**Peak hourly ICP (72 h)**
John and Colbourne (2016)	SHAM-adult	4.30	7.21	13.88	17.15	4.30	4.88	6.58	7.92
	C-ICH-adult	14.03	18.98	26.93	41.70	14.02	16.13	18.77	26.32
Hiploylee and Colbourne (2014)	SHAM-adult	2.23	4.37	9.16	15.43	2.23	4.37	4.61	9.15
	WB-I00-adult	6.06	5.34	16.09	18.28	6.06	5.34	8.11	9.72
	C-ICH-adult	9.04	9.66	19.67	25.30	9.04	9.66	11.71	14.82
Kalisvaart et al. (2022)	SHAM-aged	4.76	N/A	17.40	N/A	4.76	N/A	9.05	N/A
	C-ICH-aged	11.71	N/A	31.57	N/A	12.18	N/A	21.57	N/A
Williamson et al. (2018)	C-ICH-adult	10.74	13.06	40.48	44.02	10.64	10.29	15.80	18.58
Williamson and Colbourne (2017)	Sham-adult	3.65	3.43	14.76	17.55	3.65	3.43	5.37	5.52
	WB-100-adult	7.67	5.94	22.25	25.74	11.05	6.16	9.75	10.95
	WB-130-adult	8.75	6.24	18.63	19.29	8.75	6.23	11.79	11.79
	WB-160-adult	10.09	7.65	25.83	43.01	10.09	7.76	12.51	17.85
Overall comparison across studies	SHAM-adult	3.23	4.91	12.11	16.53	3.23	4.24	5.39	7.76
	SHAM-aged	4.76	N/A	17.40	N/A	4.76	N/A	9.05	N/A
	C-ICH-adult	10.88	13.18	29.33	36.33	10.83	11.44	14.95	18.99
	C-ICH-aged	11.71	N/A	31.57	N/A	12.18	N/A	21.57	N/A
	WB-100-adult	6.68	5.57	18.46	21.15	7.98	5.66	8.74	10.19
	WB-130-adult	8.75	6.24	18.63	19.29	8.75	6.23	11.79	11.79
	WB-160-adult	10.09	7.65	25.83	43.01	10.09	7.76	12.51	17.85

### 3.3. Brain water content

Brain water content % (representative of edema) data was collected either by blocking out a portion of the ipsilateral and contralateral hemispheres, or by dissecting out striatal, cortical, and/or hippocampal regions in each hemisphere, along with cerebellum (See Table 1 for specific study details in each case). Tissue BWC% was measured *via* the wet-dry weight method, and weight-corrected by percentage, as done previously. For the present analysis, these regional BWC% values were averaged within hemispheres to give a standardized “hemispheric” edema value, for comparison's sake across studies. The “total” magnitude of cerebral edema was determined by subtracting cerebellar brain water content values from both ipsilateral and contralateral hemispheres, then adding these values together. Here, each ICH group was compared to respective age-appropriate pooled sham groups at their respective euthanasia time point: this was at 72 h in C-ICH-Adult, Sham-Adult, and all WB-ICH groups, while C-ICH-Aged and Sham-Aged animals were euthanized at 24 h. Thus, we did not directly compare data across ages in the C-ICH model, due to the difference in euthanasia timing.

We again emphasize that BWC% is not necessarily equivalent to “true” ICH-associated edema, as these values partly derive from serum extrusion, though we may use these terms interchangeably here.

### 3.4. Statistical comparisons

All statistical analyses and figures were done using Graphpad Prism 9.5.0 (Graphpad Software, San Diego, CA). The threshold for statistical significance was set at α = 0.05, and *p* < 0.05. Figures are presented as mean ± 95% confidence intervals (95% CIs). Effect sizes are reported as the mean difference between groups ± 95% CIs. Prior to statistical testing across groups, assumptions of normality, heterogeneity, and sphericity were confirmed using the Shapiro-Wilk normality test, the F-test, the Brown-Forsythe test, and Spearman's test for heteroscedasticity, respectively, and when appropriate. Independent means were compared using one-way ANOVA or Welch's one-way ANOVA tests, depending on whether there were significant differences in standard deviation among groups; multiple comparisons follow-up testing was then done using Dunnett's T3 test. Pre-ICP event slope data was compared using two-way repeated measures ANOVA with a Greenhouse-Geissser correction, using Sidak's multiple comparisons test upon follow-up. Across groups and within animals, edema data (ipsilateral and contralateral hemispheres, cerebellum) was compared using a 2-way ANOVA with Sidak's multiple comparisons test. Lastly, correlations between ipsilateral, contralateral, and total BWC% vs. peak and average 72 h or 68–72 h ICP (by minute) were calculated *via* Pearson's R. For correlations, a threshold of *r* < 0.4 was considered poorly correlated, while values *r* > 0.4 were considered moderately correlated, and values *r* > 0.6 were considered strongly correlated.

## 4. Results

### 4.1. Exclusions

One animal (Sham-Adult) was excluded from all analyses due to apparent probe failure (e.g., negative ICP values for >25% of the recording period). One C-ICH-Adult animal was excluded from DIICP pre-event baseline and BWC% analyses due to incomplete data. Lastly, one Sham-Adult animal was excluded from BWC% due to incomplete data. Across included experiments, there were no mortalities following WB-ICH. There were two mortalities following C-ICH in adult animals within the included experiments, for an average experimental mortality rate of 9.5% within this dataset. There were also two mortalities following C-ICH in aged animals, or an experimental mortality rate of 20%. It is possible that experimental premature mortality rates may differ with age, but we cannot directly speak to this here, given that aged animals in the present dataset were only evaluated out to 24 vs. 72 h in young animals. We have evaluated ICP out to 72 h in a larger sample of aged spontaneously hypertensive rats following ICH (unpublished data, Wilkinson et al.), however, and we did not observe significant premature mortality within this cohort.

### 4.2. Intracranial pressure characterization

#### 4.2.1. Autologous whole blood model

##### 4.2.1.1. 72 h ICP comparison: Adult animals

**Figure 2:**
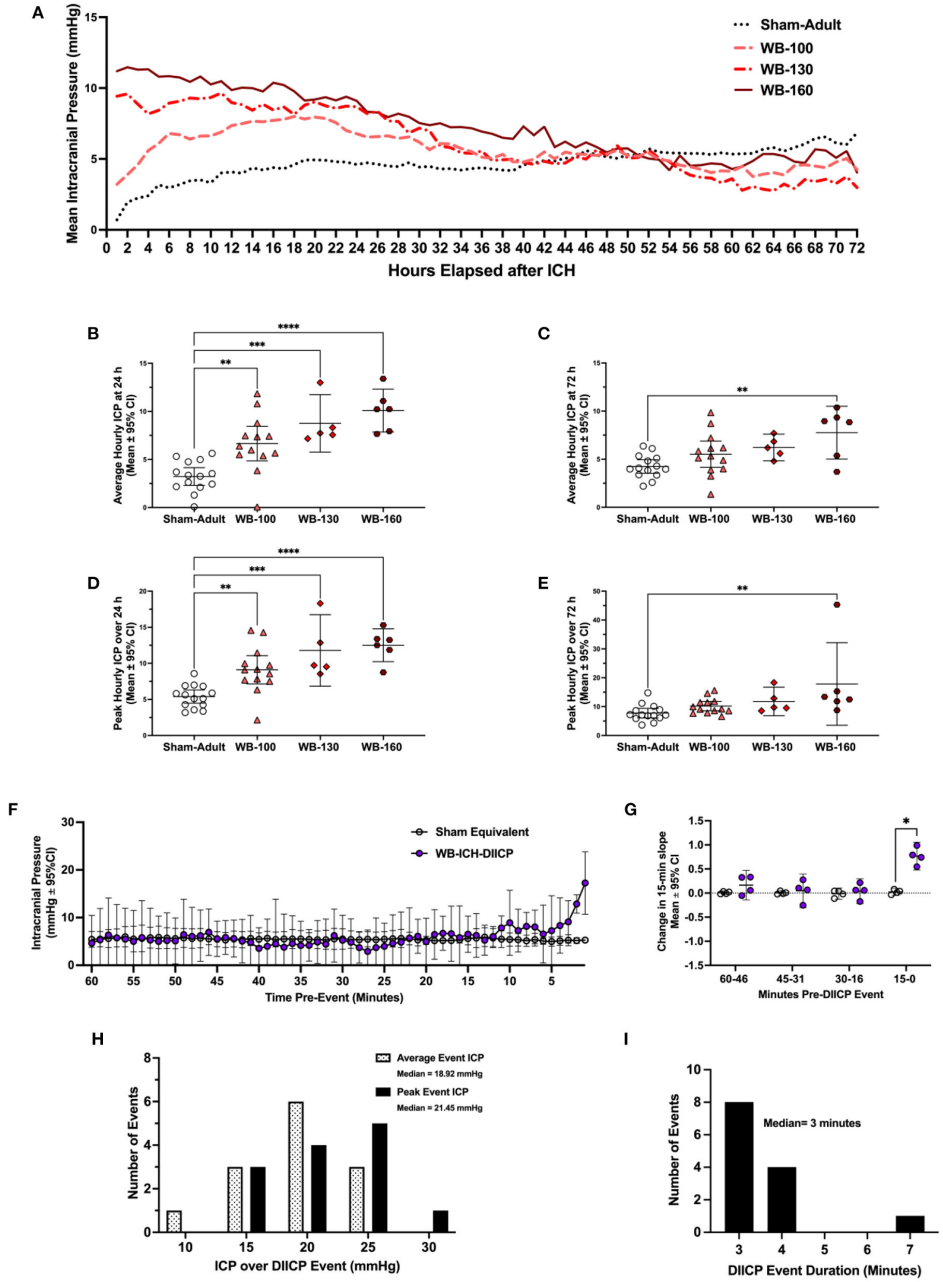
Intracranial pressure data was collected *via* telemetry following whole blood ICH (WB-ICH), or sham procedure (Sham-Adult), averaged by hour **(A)**. At 24 h post-WB-ICH, mean and peak ICP over the recording period were significantly different across all WB-ICH groups compared the Sham-Adults **(B, D)**. Average **(C)** and peak **(E)** 72 h ICP (averaged by hour) were significantly greater only in WB-160 animals compared to Sham-Adults. Pre-event ICP significantly increased in the minutes immediately prior to DIICP event onset **(F)**, as reflected by a significant non-linear increase in ICP slope in the 15 min leading up to the event **(G)**. DIICP event characteristics, such as ICP over the event duration **(H)** and event duration in minutes **(I)** are also shown. Data are presented as mean ± 95% C.I. in panels b-g, *p ≤ 0.05, **p ≤ 0.01, ***p ≤ 0.001, ****p ≤ 0.0001.

Over 24 h, there was a significant difference in both mean ICP across groups (all *p* ≤ 0.0001), where the WB-100, WB-130, and WB-160 animals all had significantly greater values compared to Sham-Adults (3.42 ± 2.43 mmHg, 5.52 ± 3.29 mmHg, 6.86 ± 3.07 mmHg, respectively, all *p* ≤ 0.005; Figures 2A, B). Peak 24 h ICP was also significantly greater across groups (all *p* ≤ 0.0001), where the WB-100, WB-130, and WB-160 animals all had significantly greater values compared to Sham-Adults (3.71 ± 2.80 mmHg, 6.40 ± 3.78 mmHg, 7.12 ± 3.54 mmHg, all *p* ≤ 0.005, Figure 2D).

Over the 72 h epoch, mean and peak ICP varied significantly by experimental group (all *p* ≤ 0.01; Figures 2C, E), but further testing demonstrated that only the WB-160 animals had statistically higher mean and peak ICP over the entire 72 h epoch vs. Shams (3.52 ± 2.46 mmHg and 10.09 ± 7.78 mmHg, respectively, both *p* ≤ 0.006).

##### 4.2.1.2. ICP spiking events

Across WB animals who experienced DIICP events (WB-ICH-DIICP), there was a significant difference in pre-event baseline ICP compared to Sham-equivalent values, where time, experimental group, and time x experimental group were significant fixed effects (all *p* ≤ 0.026; Figure 2F). Further testing demonstrating that WB-ICH-DIICP animals experienced significant ICP elevations vs. Sham-equivalent values at minute 8, 2, and 1 leading up to the DIICP event (3.03 ± 2.79 mmHg, 7.68 ± 1.85 mmHg, and 11.95 ± 6.53 mmHg respectively, all *p* ≤ 0.039). Accordingly, the local slope analysis over the pre-event baseline period indicated significant differences across groups, where time and experimental group were significant main effects, with a significant time × experimental group interaction (all *p* ≤ 0.002; Figure 2G). Further analysis revealed a significant difference in slope over the last 15–0-min period leading up to the event, where WB-ICH-DIICP animals had higher ICP slopes (*p* ≤ 0.01), but there were no significant differences over the 30–16-, 45–31-, or 60–46-min pre-event baseline epochs (all *p* ≥ 0.59). Therefore, prior to DIICP events in the WB model, there is a non-linear increase in ICP in the 15 min leading up to event initiation.

In the 24 included WB animals, there were 13 DIICP events, which occurred in four out of 24 animals (16.67%; Figure 2H). The average DIICP event duration was 3.3 min, occurring on average at 40.03 h post-ICH (Figure 2I). The average ICP over the DIICP event in WB animals was 15.75 mmHg, while the peak ICP was 17.55 mmHg (Figure 2H). There were no DIICP events that occurred prior to 24 h post-ICH.

There were no WB-ICH animals who experienced RICP events.

#### 4.2.2. Collagenase model

##### 4.2.2.1. 24 h ICP comparison: Adult and aged animals

**Figure 3:**
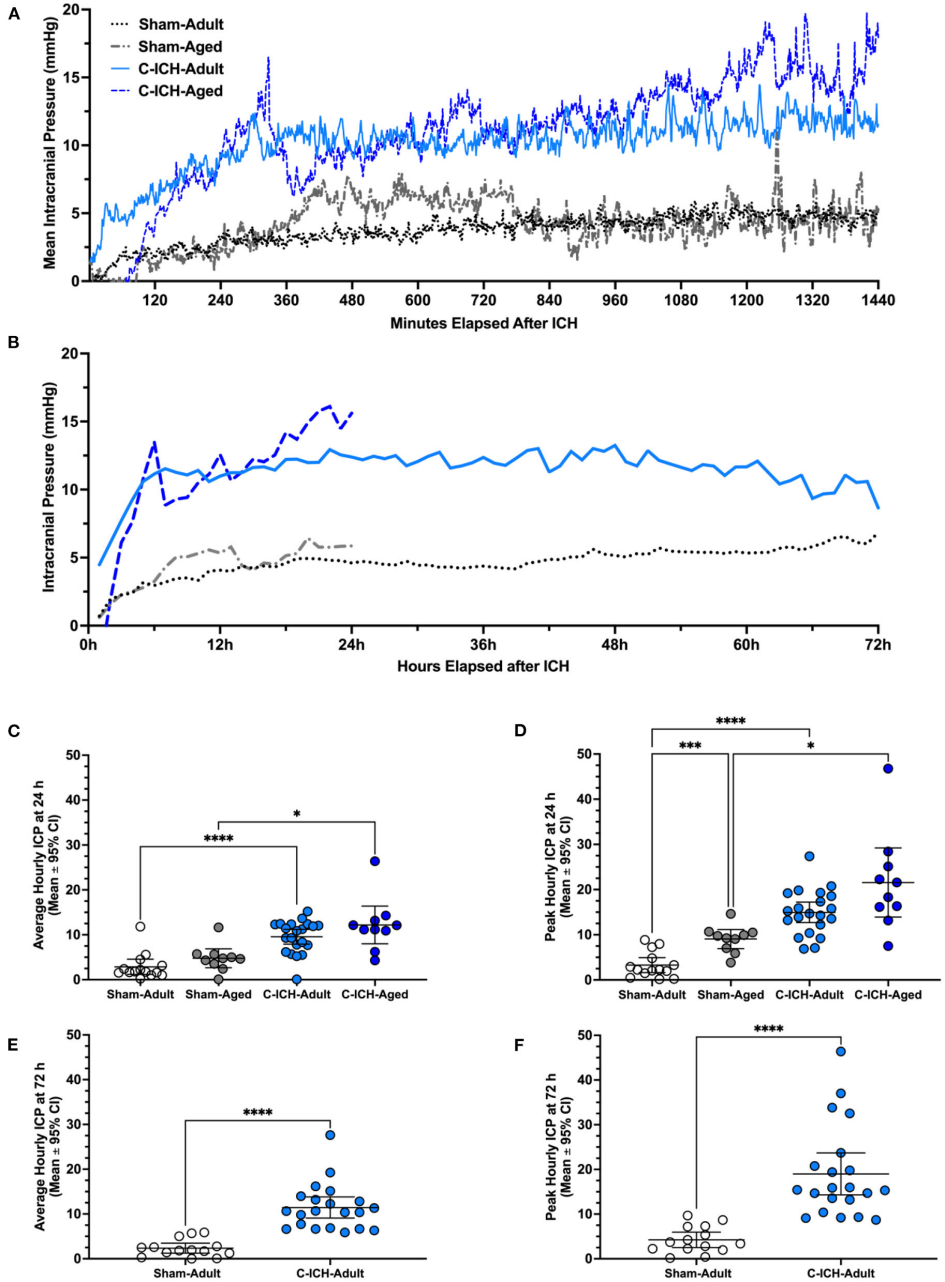
Intracranial pressure data was collected *via* telemetry following collagenase ICH (C-ICH-Adult and C-ICH-Aged) or sham procedure (Sham-Adult or Sham-Aged), averaged by minute over the 24 h epoch **(A)** and by hour over the 72 h epoch **(B)**. At 24 h post-C-ICH, mean and peak ICP over the recording period were significantly in C-ICH-Adults and C-ICH-Aged animals compared to their equivalently aged Sham counterparts **(C, D)**. Sham-Adults and Sham-Aged animals had significantly higher peak 24 h ICP **(D)**. Average **(E)** and peak **(F)** 72 h ICP (averaged by hour) were also significantly greater compared to Sham-Adults. Data are presented as mean ± 95% unless indicated otherwise, *p ≤ 0.05, ***p ≤ 0.001, ****p ≤ 0.0001.

The average ICP for the 24 h recording period varied significantly across groups (*p* ≤ 0.0001, Figures 3A–C). Further comparisons demonstrated that there was no significant difference in mean 24 h ICP between C-ICH-Adult animals and C-ICH-Aged animals (2.62 ± 5.82 mmHg, *p* ≥ 0.60), or between Sham-Adult and Sham-Aged animals (1.90 ± 3.34 mmHg, *p* ≥ 0.42), though aged animals had higher ICP on average in each case. There was a significant difference in mean 24 h ICP between C-ICH-Adult and Sham-Adult animals (6.70 ± 2.92, *p* ≤ 0.0001), as well as between C-ICH-Aged and Sham-Aged groups [7.41 ± 5.93 mmHg, *p* ≤ 0.013 (Heuts et al., 2013)].

The peak ICP over the 24 h epoch varied significantly by experimental group (*p* ≤ 0.0001, Figures 3A, B, D). Multiple comparisons testing demonstrated that peak 24 h ICP differed significantly across C-ICH-Adult vs. Sham Adult groups (11.66 ± 3.49 mmHg, *p* ≤ 0.0001), and C-ICH-Aged vs. Sham-Aged groups (12.51 ± 10.47 mmHg, *p* ≤ 0.02). There was no significant difference in peak 24 h ICP between C-ICH-Adult and C-ICH-Aged groups (6.62 ± 10.42, *p* ≥ 0.29), but there was a significant difference in peak 24 h ICP between Sham-Adults and Sham-Aged animals (5.77 ± 3.29, mmHg, *p* ≤ 0.0005). In each case, peak 24 h ICP was also higher in aged animals compared to their younger counterparts.

Therefore, C-ICH caused higher average and peak ICP in both adult and aged animals over 24 h. Sham-Aged animals had significantly higher peak, but not mean 24 h ICP compared to Sham-Adults, perhaps indicating the effect of age on dynamic ICP regulation over time. Interestingly, there was no difference in mean or peak 24 h ICP between C-ICH-Adult and C-ICH-Aged animals, though C-ICH-Aged animals had higher values in each case.

##### 4.2.2.2. 72 h ICP comparison: Adult animals

The average ICP over the 72 h recording period was significantly higher in C-ICH-Adult animals vs. Sham-Adults (9.09 ± 2.35 mmHg, *p* ≤ 0.0001; Figures 3A, B, E). The peak 72 h ICP was also significantly higher in C-ICH-Adults vs. Sham-Adults (14.75 ± 2.38 mmHg*, p* ≤ 0.0001, Figures 3A, B, F). Therefore, average and peak ICP across the 72 h recording period reached a greater magnitude in C-ICH-Adult animals compared to Sham-Adults across studies.

##### 4.2.2.3. ICP spiking events

**Figure 4:**
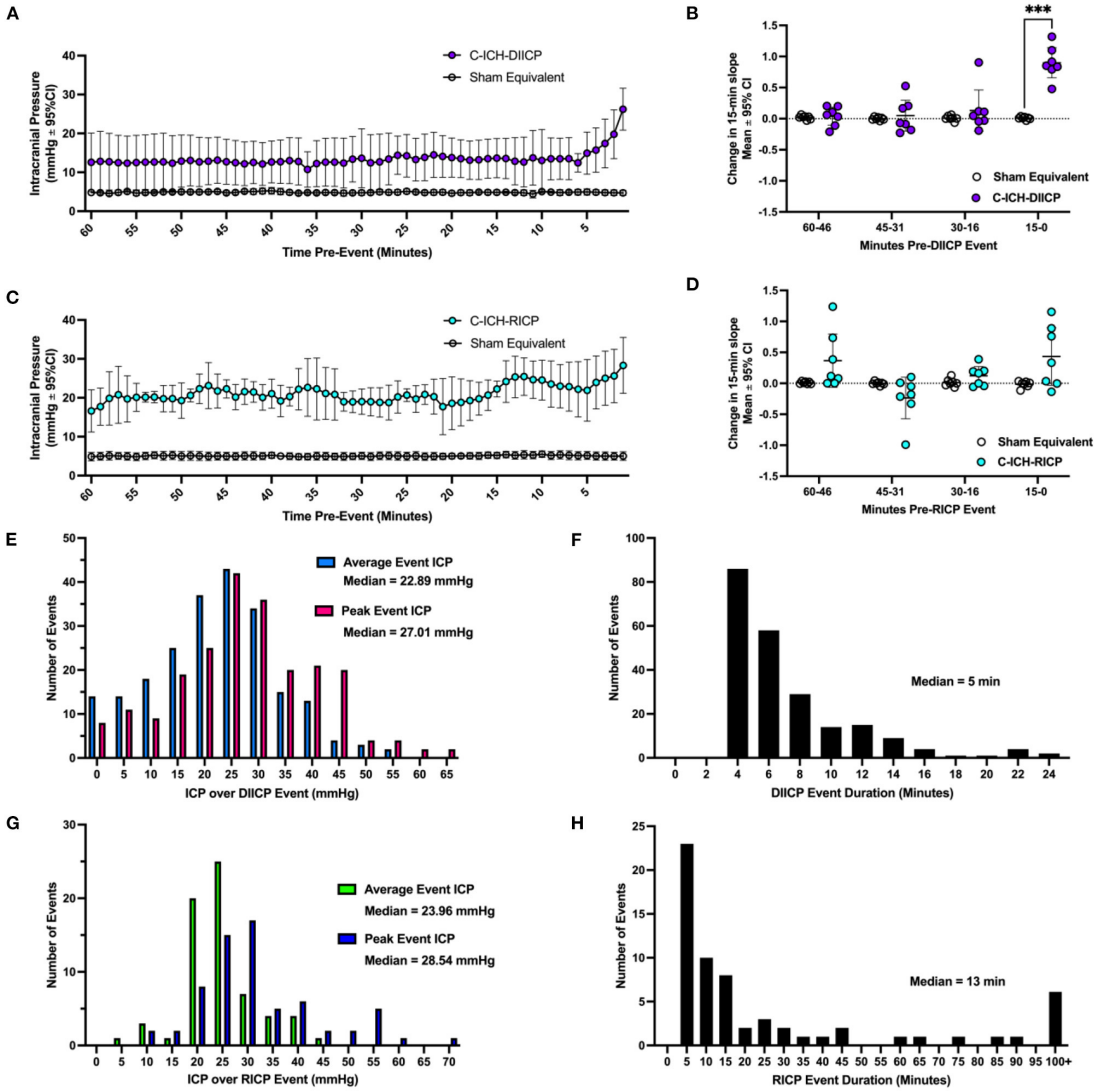
A characterization of ICP spiking events following collagenase ICH. In ICH animals who experienced ICP events (ICH-DIICP and ICH-RICP animals), the 60 min of data prior to each DIICP and RICP event was extracted, averaged within animals, and compared to equivalent sham data (e.g., “pre-event baseline period”, panels **A, C**). Prior to DIICP events, the average baseline ICP differed significantly in DIICP-ICH animals vs. Sham-equivalent values by 1-min prior to the event's initiation (**A**, *p* ≤ 0.03). Prior to RICP events, the average baseline ICP differed significantly in RICP-ICH animals vs. Sham-equivalent values over the entire 60-min period (**C**, *p* ≤ 0.001). The rate of ICP change over each 15-min epoch within the pre-event baseline period was then compared across groups for both DIICP **(B)** and RICP **(D)** events, respectively, as a measure of local ICP variability. The distribution of average and peak ICP over DIICP events are shown in **(E)**, along with the event duration **(F)**. The distribution of average and peak ICP over RICP events are shown in **(G)**, along with event duration **(H)**. ***p ≤ 0.001.

The DIICP pre-event baseline ICP varied significantly by time, experimental group, and a time × experimental group interaction (all *p* ≤ 0.008, Figure 4A). Further analyses demonstrated that ICP increased at minute 6 pre-DIICP event, and minute 1 pre-DIICP event in C-ICH-Adult animals that experienced DIICP events (C-ICH-DIICP) compared to Sham-equivalent data (14.52 ± 9.67 mmHg, *p* ≤ 0.008). The local slope analysis demonstrated that slope varied significantly by experimental group, time, and time x group interaction (all *p* ≤ 0.0002; Figure 4B), with further analysis demonstrating that the change in slope was significantly different in C-ICH-DIICP animals vs. Sham-equivalent data in the last 15 min leading up to the DIICP event (*p* ≤ 0.0004, Figure 3B). The slope over the three preceding epochs (e.g., 60–46-, 45–31-, and 30–16-min pre-event epochs) in the pre-event baseline period were all non-significant compared to Sham-equivalent data (all *p* ≥ 0.87). Therefore, DIICP events in C-ICH-Adults are characterized by a sudden non-linear increase in ICP 15 min prior to the event initiation, similar to their aged counterparts (Kalisvaart et al., 2022).

There were 225 DIICP events among 21 C-ICH-Adult animals throughout the 72 h recording period (Figure 4E). In total, six out of 21 C-ICH-Adult animals experienced DIICP events (29%). Across studies, all C-ICH-Adult animals who experienced events had more than one event, with 36 events on average per animal across those who experienced DIICP events. The mean DIICP event duration was 6.91 min long in C-ICH-Adults (Figure 4F). The average ICP over C-ICH-DIICP events was 22.29 mmHg, and the peak ICP was 27.52 mmHg (Figure 4E). For each adult ICH animal that experienced DIICP events, 15% of them happened within 24 h of their stroke, while the other 85% occurred in the 24–72 h time frame. Within the 14 Sham-Adult animals included across experiments, only one animal experienced a brief singular DIICP event.

##### 4.2.2.4. RICP events

The pre-RICP event baseline ICP varied by experimental group (*p* ≤ 0.0001; Figure 4C), but there was no significant effect of time or interaction (both *p* ≥ 0.17). The average difference in ICP over the pre-event baseline period was 16.11 ± 0.93 mmHg, greater in C-ICH-RICP animals. The effect of experimental group on pre-event ICP slope was significant, but there was no effect of time leading up to the event initiation (*p* ≥ 0.062). Similarly, there was no significant change in ICP slope over time across each four 15-min pre-event epoch in C-ICH-RICP animals vs. Sham-equivalent data (all *p* ≤ 0.25, Figure 4D), despite a significant time by experimental group interaction (*p* ≤ 0.05). Therefore, RICP events in C-ICH-Adults are characterized by a baseline period with persistently elevated ICP which continues to climb consistently over the RICP event duration, also as observed in aged animals following ICH (Kalisvaart et al., 2022).

There were 66 RICP events total detected across all 21 C-ICH-Adult animals throughout the 72 h recording period (Figure 4G). In total, 7 out of 21 C-ICH-Adult animals experienced RICP events (33%). None of the Sham-Adult rats experienced RICP events. All C-ICH-Adult animals who experienced DIICP events also experienced RICP events. There was only 1 C-ICH-Adult animal who experienced RICP events in the absence of DIICP events. Across data sets, C-ICH-RICP animals had an average of 9.57 events over the 72 h recording period. The mean ICP over RICP events in C-ICH-RICP animals was 24.74 mmHg, while the peak ICP over the event was 31.66 mmHg (Figure 4G); the average event duration was 50.63 min (Figure 4H). For each C-ICH-RICP animal, 7% of these events occurred in the first 24 h, with the remaining 93% occurring from 24 to 72 h.

### 4.3. Brain water content analyses

#### 4.3.1. Autologous whole blood model

##### 4.3.1.1. 72 h brain water content: Adult animals

**Figure 5:**
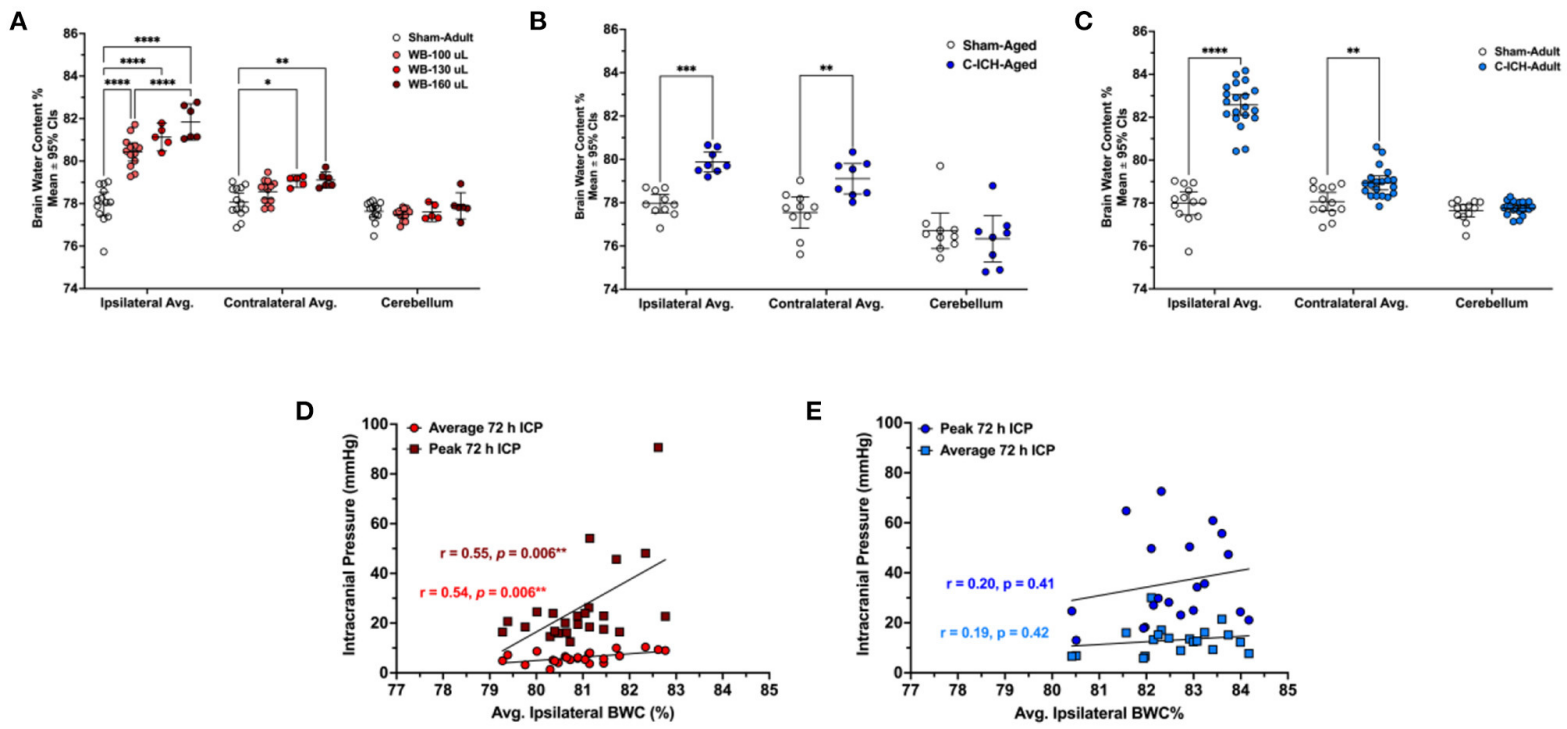
The average percentage of brain water content in the ipsilateral hemisphere, contralateral, and cerebellum in WB-ICH animals **(A)**, C-ICH-Aged animals **(B)**, and C-ICH-Adult animals **(C)** vs. equivalently aged Sham controls. The WB-ICH-Adult animals had significantly greater ipsilateral BWC% across bleed severities compared to Sham-Adults, while contralateral BWC% was only elevated in WB-130 and WB-160 animals **(A)**. In aged animals at 24 h, BWC% differed significantly across the ipsilateral and contralateral hemispheres, but not the cerebellum following C-ICH vs. age-matched controls at 24 h **(B)**. In adult animals, BWC% also differed significantly across both the ipsilateral and contralateral hemisphere, but not the cerebellum following C-ICH vs. age-matched controls at 72 h **(C)**. There were significant correlations between average 72 h ICP, peak 72 ICP, and ipsilateral BWC%, but only in the WB-ICH models when collapsing across bleed severities **(D)**. There were also no significant correlations between average or peak 72 h ICP and ipsilateral BWC% in adult C-ICH animals **(E)**. *p ≤ 0.05, **p ≤ 0.01, ***p ≤ 0.001, ****p ≤ 0.0001.

Brain water content varied by experimental group across brain regions (all *p* ≤ 0.0001; Figure 5A). Ipsilaterally, BWC% across all three WB-ICH severities were significantly different compared to Sham-Adults, with mean differences of 2.44 ± 0.62%, 3.14 ± 0.83%, and 3.86 ± 0.79% across WB-100, WB-130, and WB-160 groups, respectively, (all *p* ≤ 0.0001). There was also a significant difference in ipsilateral BWC% between WB-100 vs. WB-160 groups (1.41 ± 0.79%, *p* ≤ 0.0001), but not between WB-100 vs. WB-130, or WB-130 vs. WB-160 (0.70 ± 0.84% and 0.72 ± 0.96%, respectively, both *p* ≥ 0.14). Contralaterally, there was no significant difference in BWC% between WB-100 animals vs. Sham-Adults (0.50 ± 0.63%, *p* ≥ 0.17) but BWC% was significantly elevated in WB-130 and WB-160 vs. Sham-Adults (0.99 ± 0.84% and 1.05 ± 0.78%, both *p* ≤ 0.014). There were no significant differences in contralateral BWC% between WB-ICH groups (all *p* ≥ 0.27). Lastly, there were no significant differences in cerebellar BWC% across groups (all *p* ≥ 0.53). Therefore, there are significant elevations in ipsilateral BWC% following the WB-ICH model at all bleed sizes assessed compared to controls, while *contralateral* BWC% is only statistically detectable following larger bleeds. Cerebellar BWC% was the same across groups, as expected.

#### 4.3.2. Collagenase model

##### 4.3.2.1. 24 h brain water content: Aged animals

Brain water content varied by experimental group across regions (all *p* < 0.001; Figure 5B). At 24 h post-procedure, ipsilateral and contralateral average BWC% was significantly elevated in the C-ICH-Aged group vs. Sham-Aged controls (1.92 ± 1.11%, *p* < 0.01; and 1.56 ± 1.11%, *p* < 0.001, respectively). Cerebellar BWC% did not differ significantly between groups, as expected (0.36 ± 1.11%, *p* = 0.80). Therefore, there are significant elevations in both average ipsilateral and contralateral BWC% at 24 h post-ICH in aged male animals.

##### 4.3.2.2. 72 h brain water content: Adult animals

Brain water content varied by experimental group across all regions (all *p* ≤ 0.0001, Figure 5C). At 72 h post-procedure, average ipsilateral BWC% was significantly greater in C-ICH-Adult animals vs. Sham-Adults (4.59 ± 0.63%, *p* ≤ 0.0001). Average contralateral BWC% was also significantly greater in C-ICH-Adult animals compared to Sham-Adult controls (0.87 ± 0.63%, *p* ≤ 0.003). There were no significant differences in average cerebellar BWC% between groups, as expected (all *p* ≥ 0.95).

Therefore, there are significant elevations in both average ipsilateral and contralateral BWC% persisting out to 72 h in adult male animals following the collagenase model of ICH.

### 4.4. Relationship between ICP and brain water content

When collapsing across WB-ICH groups, there was a moderate correlation between ipsilateral BWC% and average 72 h ICP (*r* = 0.54, *p* = 0.006; Figure 5D). There was also a moderate correlation between ipsilateral BWC% and peak 72 h ICP (*r* = 0.55, *p* = 0.006; Figure 5D). The total magnitude of elevated ipsilateral and contralateral BWC% (ipsilateral + contralateral BWC%, corrected by cerebellar values) correlated moderately with average 72 h ICP (*r* = 0.50, *p* = 0.014). Additionally, there was a moderate correlation between total elevation in BWC% and peak 72 h ICP (*r* = 0.48, *p* ≤ 0.018, Figure 5D). Contralateral BWC% alone did not significantly predict ICP parameters (data not shown).

In C-ICH-Adult animals, on the other hand, there were no significant correlation between average ipsilateral BWC% and average 72 h ICP (*r* = 0.19, *p* = 0.42, Figure 5E). There was also no significant correlation between average ipsilateral BWC% and peak 72 h ICP (*r* = 0.20, *p* = 0.41, Figure 5E). Contralateral BWC% alone, along with total BWC%, did not significant predict ICP parameters both over the duration of the recording period and over the last 4 h prior to euthanasia (data not shown), which one might have expected to better reflect edema.

Therefore, in the C-ICH model, there was no appreciable linear relationships between BWC% and ICP parameters assessed. Inclusion of C-ICH-Aged animals did not notably change their association. Inclusion of Sham-Adults in these analyses resulted in significant linear relationships by broadening the range of data (data not shown). In contrast, across WB-ICH bleed severities, there appeared to be some relationship between BWC% magnitude and ICP. These significant WB-ICH relationships do not hold up when correlating the same parameters within each separate WB-ICH group, indicating that smaller differences in BWC% within groups are reasonably well-accommodated by intracranial compliance mechanisms.

## 5. Discussion

Our pooled data analysis allows us to better characterize the effect of moderate to severe ICH on ICP dynamics in the rat C-ICH and WB-ICH models, along with establishing their relationship to edema (as measured by regional BWC%). In our hands, the C-ICH model causes more severe and prolonged perturbations in average and peak ICP over time, whereas ICP perturbations are shorter and less severe in the WB-ICH model, with exception of the largest bleed severities (i.e., WB-160 animals). Both ICH models cause ICP spiking events and greater variability over time, though these are more pronounced following C-ICH; events primarily occurred over the 24–72 h period. Following C-ICH, age did not affect peak or average 24 h ICP, but aged controls had higher peak ICP values compared to their younger counterparts, indicating the impact of age on dynamic ICP regulation over time. In adult animals, both ICH models resulted in appreciable elevations in edema, but especially so in the C-ICH model. Overall, mean and peak ICP values related poorly to edema following C-ICH, while there is a significant relationship between these parameters when collapsing across WB-ICH bleed sizes, but not within groups. These data highlight the insensitivity of BWC% as a surrogate measure of ICP following ICH, especially when assessing across a relatively narrow range of severity. Inclusion of sham controls or assessment across a wide range of injury severity (producing a wide range in ICP perturbation) may produce correlations, but these often have limited predictive value overall. Ultimately, we emphasize the role of individual intracranial compliance capacity in accommodating ICH mass effect, which in our studies likely has helped keep mortality low despite substantial mass effect (bleed and edema) which seem proportionally greater (% of brain volume) than average ICH bleeds in patients (Wilkinson et al., 2021).

Of the two models, the C-ICH model appears to produce elevations in ICP that, on the surface at least, have better face validity for modeling humans with concern to post-stroke ICP dynamics. In patients, ICP elevations begin within a few hours of ICH-onset, remaining elevated for days to weeks following severe stroke (Papo et al., 1979). Similarly, we show here that both adult and aged animals experience elevated ICP that rapidly increases in magnitude from control levels over the first 6–12 h following C-ICH, following a more gradual course of hematoma formation/expansion. These ICP elevations persist out to 72 h post-ICH. The WB-ICH model, on the other hand, is characterized by immediately elevated ICP from the beginning of the recording period as a function of bleed size, and elevations do not persist out to 72 h, with exception of the worst bleed severity (e.g., WB-160 animals). The greater severity in edema and inflammation that are associated with the C-ICH model, as we have previously documented (MacLellan et al., 2008, 2010), likely contribute to these differences in ICP profile across models, aggravating and prolonging ICP perturbations. It appears that edema has a relatively larger role in driving ICP rises in the C-ICH model, whereas bleed size largely drives the degree of ICP perturbation in the WB-ICH model.

The W-ICH bleed severities used in the included studies are larger than typically used within the literature (usually ranging from 50 to 100 μL). Though bleed size could not directly be determined in the C-ICH or WB-ICH animals included in this dataset, the experiments included here along with previous studies suggest that residual 72 h bleed volumes could range from 50 to 120 μL in C-ICH animals, or an estimated ~50 to 100 μL in residual bleed volume when collapsing across WB-ICH dose severities. In our hands, a single collagenase dose meant to model moderate to severe ICH may result in bleeds of variable volumes ranging by much as 50–75 μL across animals; though residual bleed volumes are typically comparatively less variable when stratifying by WB-ICH severity, there is likely a similar range in residual bleed volume (~50 μL) when collapsing across WB-ICH group severities. In general, as a percentage of total brain size, these bleeds in rodents are larger than what is typically lethal in humans (e.g., bleeds of 50–100 μL take up 3–5% of the total average rodent brain volume, while 50–100 mL bleeds take up 2.9–5.8% of the average human brain volume) (Wilkinson et al., 2021). Although mortality was low compared to human bleeds, our bleed volumes are larger than what many use in the ICH field. It is likely then, that compliance reserves are proportionally greater in rodents than humans.

Accordingly, C-ICH-Adult animals had a comparatively higher rate of DIICP spiking events over the 72 h recording period vs. WB-ICH animals at all bleed severities. ICP spiking (DIICP and RICP events) and variability generally indicate worsening intracranial compliance (Fan et al., 2010; Williamson et al., 2019; Svedung Wettervik et al., 2020), as mechanisms such as redirection of venous blood, altered CSF flux, and brain tissue compliance become insufficient in the face of rising ICP magnitude or impaired due to injury (Wilson, 2016; Bothwell et al., 2019, 2021; Kalisvaart et al., 2020). Within each model, DIICP spiking events were proceeded by a non-linear change in ICP slope in the 15 min prior to event onset, similar to patients (Fan et al., 2010), and similar to our previous findings in aged animals following C-ICH (Kalisvaart et al., 2022). Conversely, RICP events were not proceeded by the same change in slope, as they are reflective of persistently elevated ICP exceeding 20 mmHg, also similar to our findings in aged animals (Kalisvaart et al., 2022). Adult animals experienced a greater number of ICP spiking events on average compared to their older counterparts in the 24 h following C-ICH, possibly reflecting the impact of age-related atrophy on intracranial compliance (Kalisvaart et al., 2022). Interestingly, adult WB-ICH animals did not experience any RICP events, whereas nearly all C-ICH-Adult animals who experienced DIICP events also experienced RICP events. The DIICP and RICP events in C-ICH-Adult animals occurred on average around ~44 h post-ICH, the timing of which coincides with progression of edema development in ICH models, with peak edema levels occurring at ~48–72 h post-ICH.

Edema development in the WB-ICH model can primarily be attributed to serum extrusion, with limited “true” perihematomal edema, as mentioned previously (Williamson and Colbourne, 2017), while the C-ICH model has greater disturbances in BBB integrity after stroke onset, along with worse associated inflammation (MacLellan et al., 2008; Nadeau et al., 2019). Model differences, such as BBB injury and edema development, as well as other factors, such as seizures occurring more so in the C-ICH model (Klahr et al., 2015), may underlie the disparity in ICP spiking profile between adult C-ICH and WB-ICH groups in this analysis. While WB-ICH results in limited DIICP events, indicating that the associated mass effect presents some challenge to intracranial compliance, C-ICH results in a higher rate of both types of ICP spiking events, indicating worse associated mass effect that presents a comparatively greater ongoing challenge to intracranial compliance. Across both adult ICH models, spiking events occurred primarily from 24 to 72 h post-ICH. It is interesting that most DIICP events experienced by WB-ICH animals still occurred over the 24–72 h time frame, given their more immediate elevated ICP response following autologous blood injection; this suggests that the large mass of the hematoma itself is reasonably well-compensated for, and that development of ionic and vasogenic edema along with associated inflammation may eventually overwhelm residual compliance mechanisms, leading to ICP elevations along with spiking behavior (primarily following C-ICH). Of course, as noted above, other mechanisms may be at play, such as the greater propensity for C-ICH animals to experience minor seizure activity (Klahr et al., 2015, 2016; Wilkinson et al., 2020), or perhaps a greater likelihood for subarachnoid or intraventricular bleed extension following C-ICH, especially compared to the moderate WB-ICH blood infusions (MacLellan et al., 2008); these factors may have additive and possibly non-linear effects on ICP measures.

Paradoxically, there was no relationship between average ICP, peak ICP, and any measure of edema when considering the entire C-ICH-Adult dataset. If including Sham-Adult controls, these relationships were significant, however. There was a moderate relationship between these ICP parameters and edema measures when including all WB-ICH bleed severities. These relationships were no longer significant when assessing within each individual WB-ICH group (inclusion of only WB-100 animals when correlating average ICP with ipsilateral edema, for instance, results in a non-significant Pearson's *r* of 0.22, vs. a significant *r* value of 0.54 when including all WB-ICH severity groups in the correlation). This implies that elevated cerebral BWC% and ICP only have a positive association across a wide range of injury severities with distinct and substantial differences in mass effect magnitude. Here, an *r* of 0.54 means that even across WB-ICH bleed severities, only 29% of the variance in average ICP, for example, is accounted for by edema. This also indicates that smaller changes in brain edema are reasonably well-accommodated by intracranial compliance mechanisms at moderate stroke sizes (e.g., 100 μL in WB-ICH model and ~75 μL bleed in C-ICH model). When considering only animals who experienced ICP spiking events, the number of ICP spiking events in each ICH animal who experienced them correlated significantly to BWC% measures (data not presented), but there was not a large enough sample size to reliably establish these relationships, as ICP spiking only occurs in ~1/3 of all ICH animals (likely those with a trifecta of insufficient compliance reserves along with severe bleeds and a greater degree of cerebral edema). Closer assessment of the relationship between ICP spiking, intracranial compliance reserves, and edema remains an important direction to explore in the future.

A major limitation of *in vivo* studies of intracranial compliance following stroke and brain injury is that it is difficult to reliably measure all compliance mechanisms in the same animal. In a preclinical ICH study, for example, this would necessitate simultaneous assessment of ICP, hematoma volume, edema progression, brain volume, cerebral autoregulation, blood pressure, and CSF flux, all of which are dynamic parameters that change and evolve both temporally and spatially. Unfortunately, this is not currently feasible, though computational modeling approaches have been attempted (Kim et al., 2012; Vinje et al., 2020), and wider availability and technological capability of high-resolution small animal imaging devices have improved data throughput somewhat in this regard (Driehuys et al., 2008), along with refinement of implantable telemetry devices. Blood pressure does not seem to markedly rise in these rat models of ICH, nor does CPP seem to markedly drop (Maclellan et al., 2004; Hiploylee and Colbourne, 2014; Wilkinson et al., 2020). For the most part, current approaches still suffer from an incomplete picture regarding the sequence of how intracranial compliance dynamics unfold both temporally and spatially, and how key translational parameters such as age, sex, or co-morbidity shape how these mechanisms are engaged. Additionally, anesthetic confounds are a concern within many studies. Across ICH studies more broadly, there are misconceptions regarding intracranial pressure-volume relationships and the variable capacity for intracranial compliance in rodent models of ICH. Our data here, for example, demonstrate that the use of BWC% as a surrogate marker for the degree of mass effect and ICP perturbation without considering a wider range of injury severity is misguided, and should not be use as a sole primary endpoint. The corollary here is that statistically significant changes in edema may have no discernable impact on ICP. Therapies causing modest reductions in edema, therefore, are unlikely to notably affect ICP, and so any concomitant improvement in behavior likely arise from other mechanisms (e.g., attenuation of ionic dyshomeostasis in perihematomal tissue) (Williamson et al., 2017; Nadeau et al., 2019).

As discussed, estimates of ICP can vary widely across both clinical and preclinical studies, highly dependent on the method used. Our approach, which uses telemetry probes to measure epidural ICP in awake freely moving animals, has both strengths and weaknesses. Methodological strengths of this method include lack of anesthetic confound and ability to continuously measure ICP, while drawbacks include the invasive nature of the implant and potential for movement artifacts (Silasi et al., 2009). Having the implantation procedure and probe placement consistent across all five studies (spanning over nearly a decade), helps ensure the validity of pooling these datasets while speaking to the ICP response following two rodent models of ICH with some degree of confidence in our conclusions. Our analyses had some limitations, in addition to the retrospective nature of the present analysis; namely, these included the lack of data in aged animals following WB-ICH, and data extending out to 72 h in aged animals following C-ICH. Additionally, we were unable to evaluate total bleed size across C-ICH groups, as brain tissue was allocated to BWC% analyses in each included study; therefore, we could not directly speak to the relationship between bleed size, ICP, and edema within the same C-ICH animals. We did not include data across both sexes or those with co-morbidities, but initial analyses in female (Kalisvaart et al., unpublished data) and hypertensive rodents (Wilkinson et al., unpublished data), respectively, have indicated a similarly perturbed profile of ICP following C-ICH. Establishing how these clinically relevant demographic differences affect ICP, edema, and intracranial compliance following ICH remains an important future direction.

Lastly, it is important to note that the *a priori* rationale to include only studies conducted by a single lab group in our analyses may increase the likelihood of bias, limiting generalizability of findings. The decision to include studies from only our lab group was based on a paucity of ICP data collected in preclinical rodent models of severe ICH from other groups. This also allowed us to ensure methodological consistency across our dataset, reducing the need for numerous subgroups, and maximizing statistical power to detect smaller effects. Although the five studies included originate from the same lab group, they were conducted by four different experimenters, including surgical procedures. It is also important to again note that telemetric ICP recordings are subject to some limitations. Namely, these include the impact of body position on ICP, the margin of measurement error, and the lack of control or monitoring of important clinical physiological variables, such as respiration rate, pO_2_, and pCO_2_ (Silasi et al., 2009). Additionally, ICH patients are often mechanically ventilated under the influence of anesthetics, which could be presented as an argument for using alternative ICP monitoring methods, such as *via* ventricular catheter and external pressure transducer. This warrants comparative study in future preclinical ICH studies to further improve upon clinical relevance.

In conclusion, while both models of ICH produce appreciable ICP perturbations, the C-ICH model appears to better model more clinically relevant perturbations in at-risk patients. To have 80% statistical power to detect a 25% change in average ICP in adult animals following C-ICH (two-tailed test, α = 0.05), our dataset suggests that one would need 42 animals per group, assuming consistent standard deviation across groups. This speaks to the limited practicality of establishing treatment effects using currently available telemetric ICP sensors. There was no reliable relationship between ICP parameters assessed and edema as measured by bulk assessment of regional BWC% unless considering a broad range of injury severity. Therefore, we recommend that future preclinical ICH studies should directly assess mass effect and intracranial compliance *via* continuous assessment of ICP when relevant, rather than solely relying on regional BWC% evaluation. Additional recommendations include assessment of cerebral blood flow and brain tissue oxygenation, to establish whether ICP elevations have resulted in local cerebral blood flow impairments (rather than assuming that worse cerebral edema is equivalent to more severe mass effect in all individuals). Through leveraging existing methods and techniques, the interplay of mass effect and intracranial compliance mechanisms which drive ICP dynamics can be better and more completely characterized after preclinical ICH. This ultimately will aid in improving our understanding of mortality and morbidity in patients with elevated ICP, contributing to the development of desperately needed therapeutic options for this diverse clinical population.

## Author contributions

AK and FC were involved in conceptual planning and design of retrospective analyses. AK and NB were responsible for data collation across studies, artifact removal from raw data, and running data through python code. AK and FC were involved in data analysis, while AK was responsible for making figures, and NB was responsible for formulating tables. AK and FC drafted the manuscript, which all authors edited and approved. All authors contributed to the article and approved the submitted version.
